# Divergence and Selection in a Cryptic Species Complex (*Geonoma undata*: Arecaceae) in the Northern Andes of Colombia

**DOI:** 10.1093/gbe/evaf130

**Published:** 2025-07-22

**Authors:** Carmen P Webster, Margot Paris, Ingrid Olivares, Martin F Wojciechowski, Michael Kessler, María José Sanín

**Affiliations:** School of Life Sciences, Arizona State University, Tempe, AZ, USA; School of Mathematical and Natural Sciences, Arizona State University, Glendale, AZ, USA; Department of Biology, Unit of Ecology & Evolution, University of Fribourg, Fribourg, Switzerland; Department of Systematic and Evolutionary Botany, University of Zurich, Zurich, Switzerland; School of Life Sciences, Arizona State University, Tempe, AZ, USA; Department of Systematic and Evolutionary Botany, University of Zurich, Zurich, Switzerland; School of Mathematical and Natural Sciences, Arizona State University, Glendale, AZ, USA; Collections Department, Montgomery Botanical Center, Coral Gables, FL, USA

**Keywords:** Arecaceae, genetic differentiation, genomic divergence, selection, allopatry, Tropical Andes

## Abstract

Palms (family Arecaceae) are integral to understanding the evolution of tropical rainforests due to their long evolutionary history, high species richness, and hyper dominance in these ecosystems. Some palm genera, like *Geonoma*, are regionally and locally species-rich and abundant in Neotropical rainforests, but factors contributing to their divergence and ultimately their diversification remain poorly explored. A recent phylogenomic study identified the *Geonoma undata* complex, with high levels of genetic distinctiveness of different geographically proximal groups, describing it as a hyper-cryptic radiation. Here, we seek to disentangle the factors that contribute to genetic divergence in the *G. undata* cryptic species complex in the Northern Colombian Andes, where various forms ascribable to different taxonomic, morphological, and genetic groups exist. To address this, we pursued three main aims using nuclear single nucleotide polymorphisms distributed along over 4,000 genomic regions from 156 individuals. (i) We identified populations and used diversity metrics to understand evolutionary scenarios across pairwise comparisons of those populations. Geographically sympatric populations display evidence for allopatric selection that is likely explained by elevational segregation. (ii) Tajima's D was used to infer broad genomic trends in selection and drift. In general, divergence between populations is enhanced by drift through population expansions. (iii) Lastly, we used outlier divergence and selection statistics to identify genes with outstanding divergence under significant positive selection. Two genes were identified that fit this description and are found to play functional roles in phenology, such as light response and flowering time.

Significance The underlying processes that contribute to species radiations, especially in cryptic groups of plants, where multiple morphological forms exist under different levels of genetic divergence, are unknown for many plant groups. The Andean complex comprising *Geonoma undata* is abundant in Neotropical cloud forests, and was recently shown to be hyper-cryptic with different genetic groups that do not match morphological assessments. Here we show that geographic and elevational isolation, genetic drift through population expansions, purifying selection, and positive divergent selection in phenology-related genes are all contributing to the differentiation of genetic groups within the Northern Andes. This work contributes to understanding the mechanisms underlying cryptic diversity in the highly biodiverse context of the Andes.

## Introduction

Understanding how and by which factors species diverge is fundamental to our understanding of the origin and maintenance of biodiversity. It also contributes to unraveling why some areas in the world harbor disparately high biodiversity, such as the Neotropical mountain forests, the focus area of this work. The speciation continuum is the process by which populations genetically diverge until they reach reproductive isolation (Stankowski and Ravinet 2021) with one population on one end of the continuum and two or more separate species on the other end, and studying this process can unveil the factors influencing speciation (Stankowski and Ravinet 2021). A fundamental step in studying speciation is understanding which populations are under divergence, where divergence is more marked in the genome and if there are any associated selection patterns in these differentiating populations.

Species divergence as a continuous process is particularly important in recently diversified groups where there is disparity between the species proposed by morphological and genomic assessment or where population differentiation is cryptic. Although attempting to group organisms by morphology has historically been the main method of defining species, it can lead to inaccuracy and disagreements when applied to species concepts (Galtier 2019). The recent diversification of some taxa has led to either remarkably high but inconsistent morphological diversity, or to the lack of clear morphological distinctions and inconsistencies across different spatial scales. By incorporating genomic data, taxonomists are faced with the decision of either splitting into more and more species that seem morphologically cryptic, or to clump genetically divergent populations under one big umbrella species, failing to name the actual evolutionary units. Moreover, they are faced with the question of what evolutionary processes give rise to these disparities. Because providing evolutionarily sound accounts of regional diversity in megadiverse regions like Neotropical mountains is not trivial (Stropp et al. 2022), studying taxa undergoing the process of speciation in these geographic regions should be a common priority.

Phylogenomics provides a backbone and a starting point in the selection of focal species complexes in which the speciation process and its underlying factors can be studied. Recently, Loiseau et al. (2019) used approximately 4,000 genomic coding regions out of which 795 genes were selected to construct a phylogenetic tree of palms in the tribe (taxonomic classification between subfamily and genus) Geonomateae, sampling ∼85% of traditionally recognized species in the tribe. This study unveiled a species complex consisting of the four morphologically distinct species *Geonoma undata*, *Geonoma orbignyana*, *Geonoma talamancana*  *Grayum*, and *Geonoma lehmannii* Dammer ex Burret which do not represent separate genetic units (Loiseau et al. 2019). Furthermore, a study using 130 targeted genes in 58 localities of *G. undata* and *G. orbignyana* covering the three mountain ranges of Colombia identified four genetic clusters related to geography and not to taxonomic classification (Sanín et al. 2022). More recently, Olivares et al. (2024), using 419 samples from Mexico to Bolivia, confirmed that species delimitation of these palms based on morphology does not accurately represent current genetic diversity throughout their entire geographic range. This study estimated over 11 genetically distinct groups, a process they describe as hyper-cryptic speciation, where genetic groups have likely formed to fill distinct ecological niches (Olivares et al. 2024). This species complex is hereafter referred to as the *G. undata* complex.

**Fig. 1.**
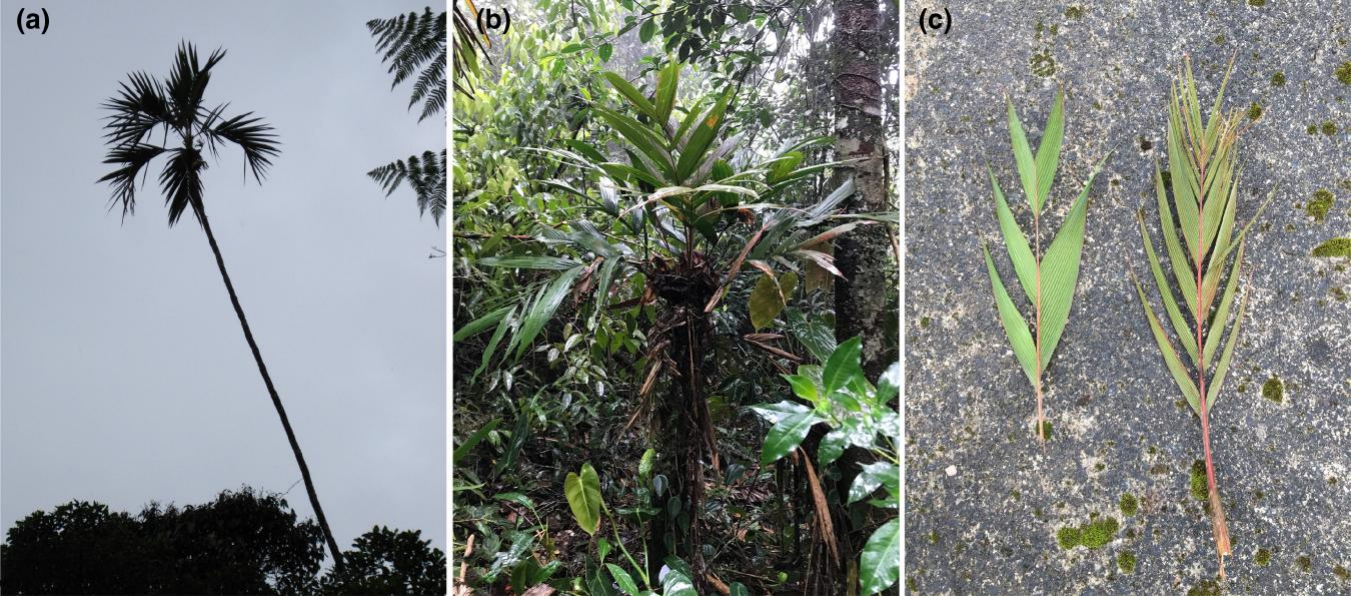
Morphological variation and complexity of the two traditionally recognized species in the *G. undata* complex. a) *Geonoma undata* in the Western Cordillera of Colombia; b) *Geonoma orbignyana* in the Central Cordillera of Colombia; c) an example of the varying leaf morphology in the *G. undata* complex in the Colombian Massif (photographs by María José Sanín).

In the Northern Colombian Andes, the *G. undata* complex is traditionally considered to be represented by the two widespread species *G. undata* and *G. orbignyana* (Loiseau et al. 2019) (Fig. 1). Both are mid-elevation to highland species that grow in all three cordilleras where *G. undata* prefers elevations between 1,200 and 3,200 m and *G. orbignyana* prefers 900 to 2,900 m (Galeano and Bernal 2010). They are long lived and can reach approximately 120 years of age (Rodríguez-Buriticá et al. 2005). *Geonoma undata* often reaches 8 to 10 m in height, whereas *G. orbignyana* is a relatively small understory palm of 2 m (Rodríguez-Buriticá et al. 2005). *Geonoma* palms are pollinated by insects, usually bees, beetles, or flies (Henderson 2011), and *G. undata* is likely pollinated by either Mystropine beetles (Knudsen 2002) or flies. *Geonoma undata* and *G. orbignyana* are solitary palms that both come in multiple morphological forms which are sometimes referred to as subspecies (Galeano and Bernal 2010) and at least partly coincide with genetic groups, raising questions about the factors that could be driving divergence in the complex. Sanín et al. (2022) found that the current phylogenetic grouping of the *G. undata* complex in the Colombian Andes follows the complex topographic history of mountain buildup and fragmentation, and that topographic gaps caused by geological faults have likely been the primary cause of divergence with dispersal by gene flow in the complex. Moreover, some of the genetic groups show completely sympatric distributions, suggesting strong reproductive isolation (Sanín et al. 2022).

Disparity in morphological and genetic divergence gives rise to different questions about the underlying genomic processes driving the diversification in the *G. undata* complex. In particular, there is a need to identify functional regions in the *G. undata* complex genome that are driving divergence between the populations. To address these questions, we combined extensive population sampling of the *G. undata* complex in the Northern Colombian Andes, including known sympatric populations, together with comprehensive genomic data comprising more than 4,000 genomic markers distributed across the entire genome. Using population genomics, differentiation genomic scans (D_XY_ and Hudson's F_ST_), nucleotide diversity, and selection estimates (Tajima's D and d_N_/d_S_), we aimed to (i) uncover which evolutionary scenarios are most likely acting in the speciation process between our pairs of genomic groups (divergence with gene flow, allopatric selection, recurrent selection or balancing selection), (ii) identify broad genomic trends of selection and drift, and (iii) detect genomic regions under divergent selection between the different genomic groups, including allopatric and sympatric pairs and understand the functional roles of these regions.

## Results

A total of 2,568,396 high quality-filtered sites, including 212,888 variable sites, were recovered across all 4,184 genic and nongenic regions in the PopcornPalm kit for the 156 individuals used in this study.

### Genetic Structure, Population Characterizing, and Phylogenetic Relationships

Once assessing Admixture cross-validation statistics, we selected ancestry coefficient K = 5 to represent these data. Of the 156 individuals in this *G. undata* complex dataset, 29 were admixed and 127 individuals displayed pure ancestry (Fig. S1). The five populations were distributed across the Eastern Cordillera (EC), the Colombian Massif (CM), and three sympatric groups in the Western and Central Cordilleras (WCC1, WCC2, WCC3) (Fig. S1). From here forward, these populations are represented by the following colors throughout the manuscript: EC is orange, CM is blue, WCC1 is pink, WCC2 is purple, and WCC3 is green. The Principal Components Analysis resulted in PC axes 1 to 3 accounting for 26.4% of genetic variance. PC axis 1 split the Colombian Massif (CM) population from the remaining four populations (Fig. S1), while axis 3 split the Eastern Cordillera (EC) population from the remaining four populations (Fig. S1), with the exception of the highly admixed MK891 individuals (Fig. S1).

### Elevational Exploration of Populations

**Fig. 2.**
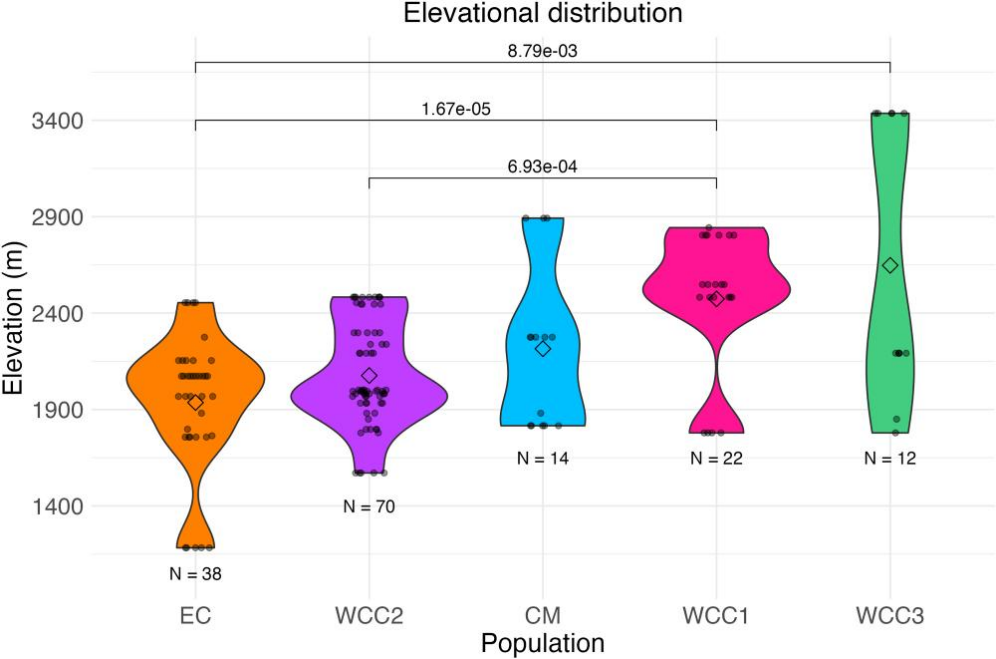
Violin plot of elevations of samples in the *G. undata* complex in the Northern Colombian Andes; each violin represents one genetic population. The diamond in each violin is representative of the mean elevation, and the total individuals per population are printed below each violin. Populations are ordered by mean elevation. Significantly different elevations are represented by brackets, along with the respective *p*-values above the brackets.

Some populations of the *G. undata* complex are located at elevations in the Northern Colombian Andes that are statistically significant from one another (Kruskal–Wallis *p* = 5.951e-06, alpha = 0.05). The pairwise comparisons between population genetic divergence (Dunn test) were significant for WCC3 and EC (*p* = 8.79e-03), for WCC1 and EC (*p* = 1.67e-05), and for the WCC1 and WCC2 (*p* = 6.93e-04) (Fig. 2).

### Nucleotide Diversity, Absolute, and Relative Divergence of Populations

**Fig. 3.**
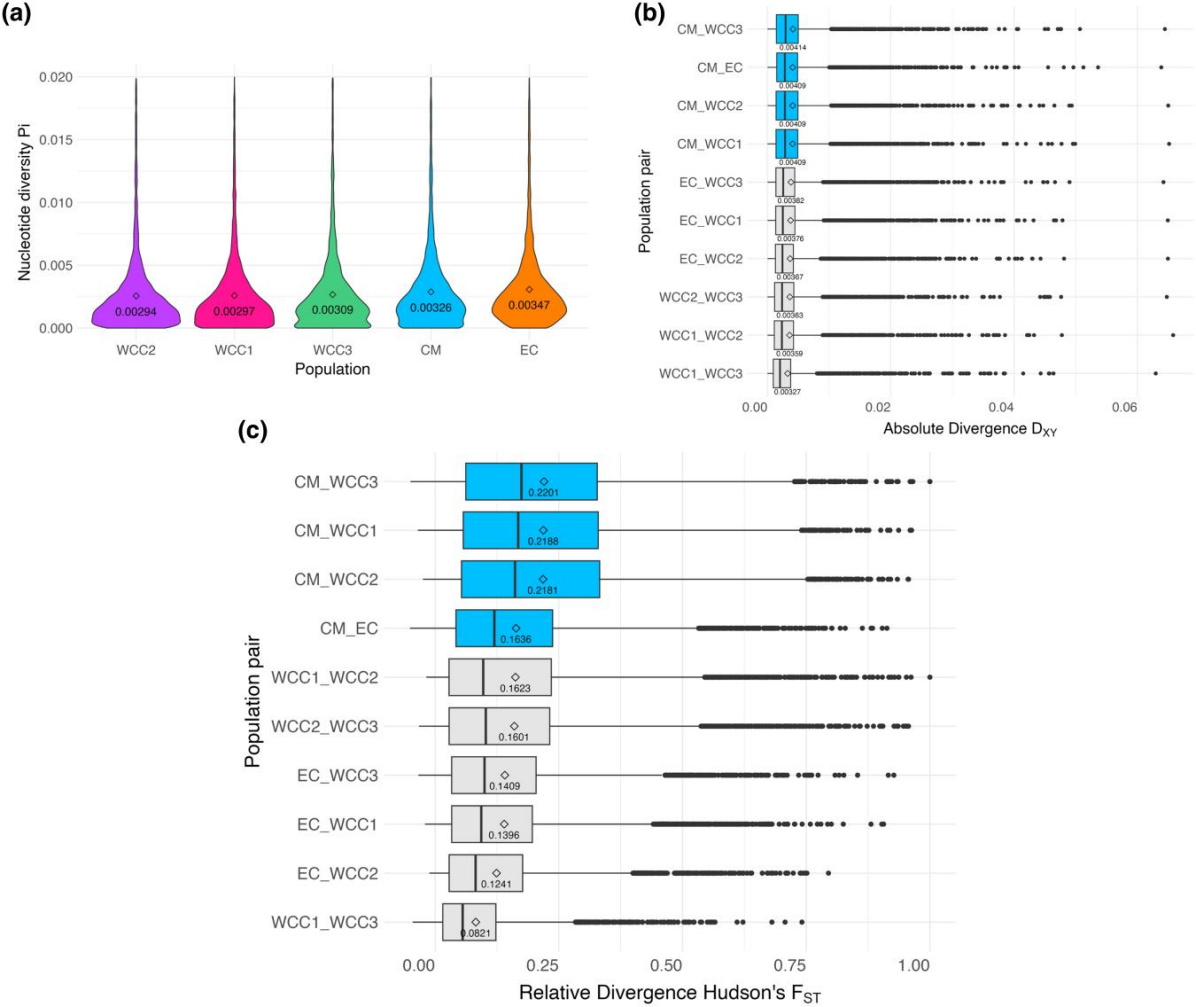
Summary statistics of genetic diversity and divergence in the *G. undata* complex in the Northern Colombian Andes. a) Within-group nucleotide diversity, Pi, of all five populations. The diamonds and numeric values in each violin plot are representative of the mean of each population. b) Between-group absolute divergence (D_XY_) values for all ten pairwise comparisons of the five populations, calculated by sliding window. Numerical values under each boxplot are representative of the mean, and the top 4 boxes in the boxplots contain pairwise comparisons with the Colombian Massif (CM) population. These represent the pairwise comparisons with the highest D_XY_ means. c) Between-group relative divergence (Hudson's F_ST_) values for all ten pairwise comparisons of the five populations, calculated by sliding window. The mean values are depicted by the diamonds in the boxplots, and their corresponding numerical value is displayed under each diamond. The top 4 boxes are representative of pairwise comparisons that contain the Colombian Massif (CM) population and contain the highest mean values for Hudson's F_ST_.

Between-group absolute divergence (D_XY_) was generally slightly higher than the within-group nucleotide diversity (Pi). We found the lowest numbers of Pi in the three WCC populations (purple: 0.00294, pink: 0.00297, and green: 0.00309) (Fig. 3a). The EC population had the highest Pi at 0.00347 (Fig. 3a). D_XY_ displayed overall low values as well (all mean values less than 0.005) (Fig. 3b). The highest mean D_XY_ were all pairwise comparisons including the Colombian Massif population, with the highest mean between the CM and WCC3 population pairwise comparison, at 0.00414 (Fig. 3b). The pairwise comparison that resulted in the lowest D_XY_ mean value was between the populations of WCC1 and WCC3, at 0.00327 (Fig. 3b). For between-group relative divergence (Hudson's F_ST_), similarly to D_XY_, we see the highest mean F_ST_ statistics for all pairwise comparisons containing the CM population, where the Colombian Massif is located, with the highest mean between the CM and WCC3 population pairwise comparison, at 0.2201 (Fig. 3c). The pairwise comparison that resulted in the lowest F_ST_ mean value was between the WCC1 and WCC3 populations, at 0.0821 (Fig. 3c).

### Comparisons of Within-Population and Between-Population Summary Statistics

**Fig. 4.**
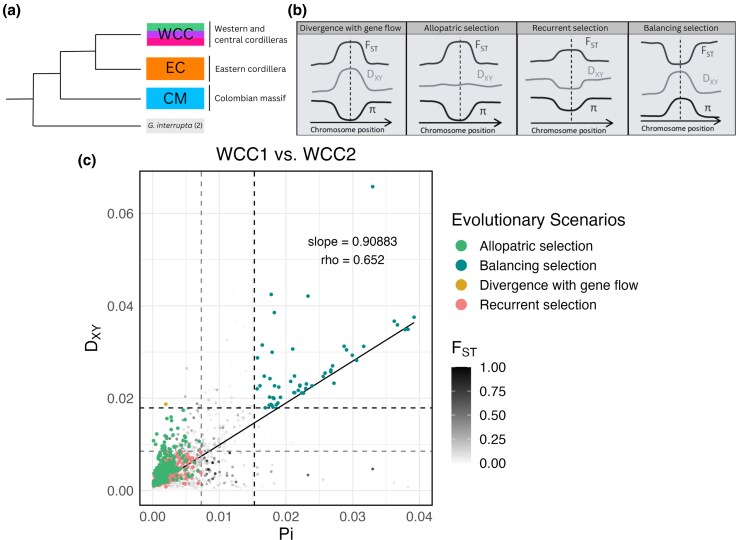
Comparison of summary statistics in two sympatric populations. a) Summary phylogenetic tree derived from Sanín et al. (2022) to display the broad relationships of populations in the northern Colombian Andes. b) Expected behavior of summary statistics and the corresponding evolutionary scenarios. This image was adapted from Han et al. (2017), Irwin et al. (2018), and Shang et al. (2023). c) Plotted summary statistics for the WCC1 versus. WCC2 pairwise comparison with a regression line in solid. Absolute divergence, D_XY_, is plotted on the *y* axis, and averaged pairwise nucleotide diversity, Pi, is plotted on the *x* axis. The upper boundary for both D_XY_ and Pi is plotted on the dashed line and is representative of three standard deviations from the mean for both statistics. The lower boundary for both D_XY_ and Pi is plotted on the dashed lighter line and is representative of one standard deviation from the mean for both statistics. The plotted points are shaded by their F_ST_ values, where a value of 1 is light and a value of 0 is dark, or they are indicated by evolutionary scenario (allopatric selection, balancing selection, divergence with gene flow, and recurrent selection). For a raw plot with no evolutionary scenarios indicated, see Fig. S2.

Overall, in the three pairwise comparisons that were studied, we found high amounts of windows displaying allopatric selection (total windows = 690) and recurrent selection (total windows = 1,569), and significantly fewer windows that display divergence with gene flow (total windows = 7) and balancing selection (total windows = 167) (Table 1). Comparison of D_XY_ and Pi for all three pairwise comparisons resulted in a positive correlation (WCC1 vs. WCC2 slope = 0.90883 and rho = 0.652, WCC2 versus EC slope = 0.98501 and rho = 0.681, and CM versus EC slope = 0.93752 and rho = 0.647). Overall, windows with high F_ST_ values tended to display allopatric selection as opposed to divergence with gene flow (Fig. 4c, Table 1).

**Table 1:** Number of windows that correspond with the different evolutionary scenarios by pairwise comparison, along with the total number of windows analyzed

Pairwise comparison	Divergence with gene flow	Allopatric selection	Recurrent selection	Balancing selection	Total windows
WCC1 versus WCC2 (sympatric)	1	242	513	57	3,969
WCC2 versus EC (allopatric)	1	234	533	61	4,103
EC versus CM (allopatric)	5	214	523	49	3,971

Evolutionary scenarios are depicted by summary statistic behaviors adapted from Shang et al. (2023), Han et al. (2017), and Irwin et al. (2018): Divergence with gene flow is represented by high F_ST_ with high D_XY_ and low Pi, allopatric selection is represented with high F_ST_ and low Pi, recurrent selection is slightly increased F_ST_ with slightly decreased D_XY_ and Pi, and balancing selection is low F_ST_ and high D_XY_ and Pi.

### Recent Population Dynamics and Concordant Genes With Positive Selection and Outstanding Divergence

**Fig. 5.**
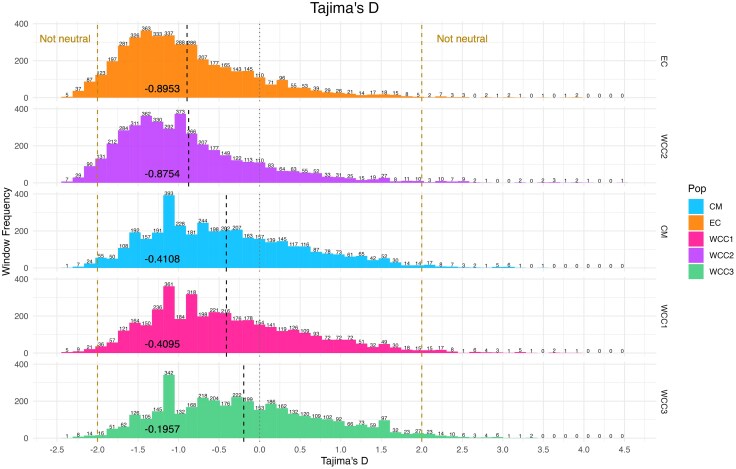
Histograms displaying the summarized Tajima's D statistics for the five populations. The dashed vertical black lines and numbers in the colored portion of each population represent the mean Tajima's D statistic per population. The lower and upper limit dashed lines show the windows of genetic regions that are likely not evolving neutrally, with Tajima's D values of either less than −2 or greater than 2. The dashed mid-line represents neutrally evolving regions with a Tajima's D value of 0. Numerical values on top of each bin in the histograms are representative of the number of windows that fall in that respective bin.

**Fig. 6.**
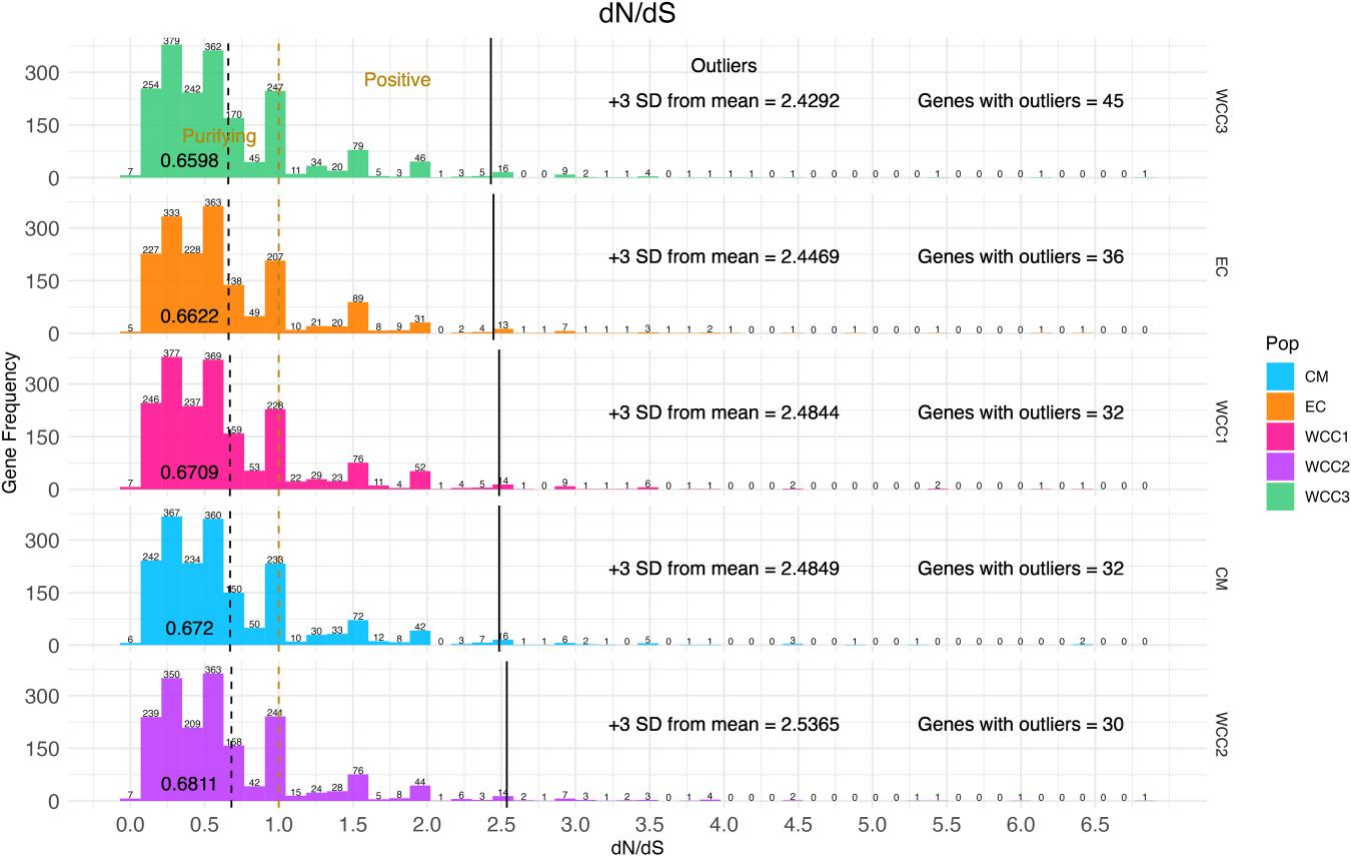
Histograms that display the summarized d_N_/d_S_ statistics for the five populations. The *x* axis displays the d_N_/d_S_ values, and the *y* axis displays the number of occurrences per gene. The dashed vertical dark lines and the adjacent numbers show the mean d_N_/d_S_ statistic per population. The upper limit dashed lines separate the windows of genetic regions that are likely under purifying selection, with values between 0 and 1, or positive selection, with values greater than 1. The solid line in each population is representative of the outlier cut off of 3 standard deviations from the mean. Windows that fall to the right of the solid lines are considered to have outlier d_N_/d_S_ statistics. The text on the right of each histogram depicts the values for the outlier cutoffs per population and the number of genes that contain windows with outlier d_N_/d_S_ statistics. Numerical values on top of each bin in the histograms are representative of the number of genes that fall in that respective bin.

**Fig. 7.**
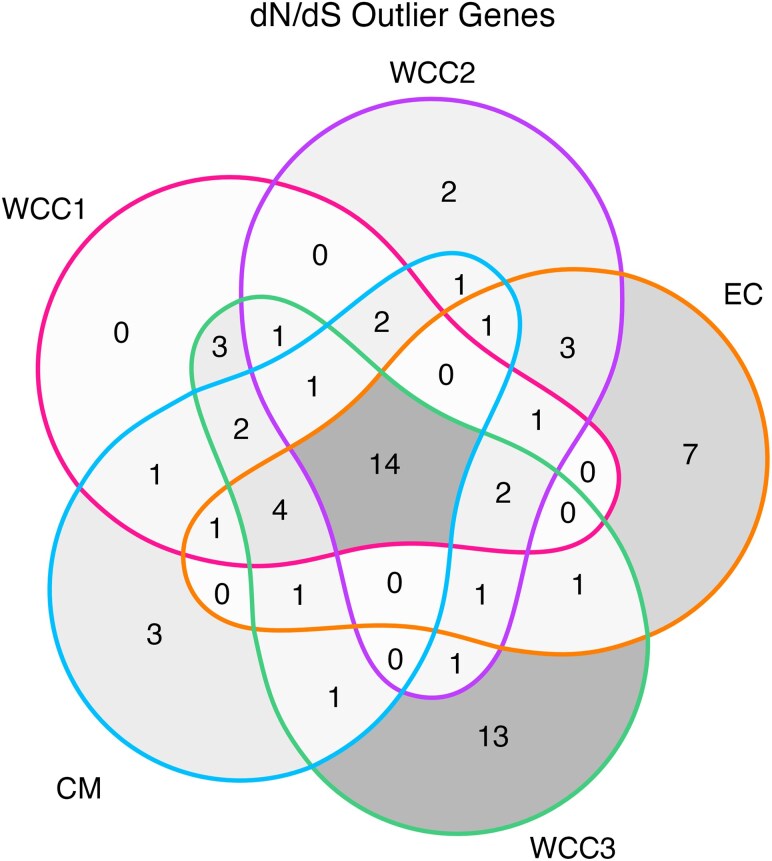
Venn diagram displaying the number of genes with outlier d_N_/d_S_ statistics per population and where those genes appear in multiple populations. Regions are shaded by gene density, where a darker shade is representative of more genes.

**Fig. 8.**
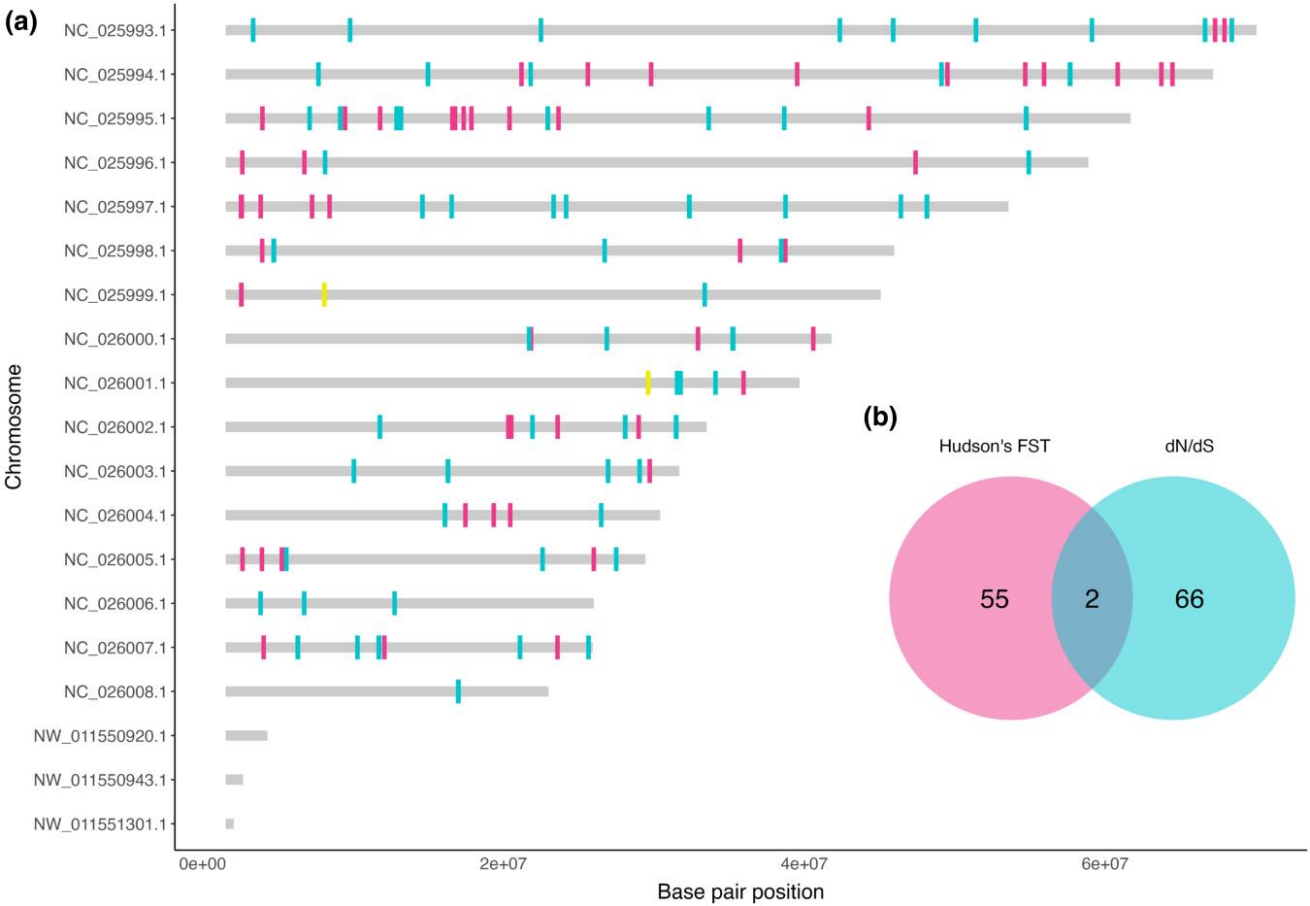
a) Locations of genes with outstanding divergence, depicted by outlier Hudson's F_ST_ values and significant positive selection, depicted by outlier d_N_/d_S_ values. These genes are mapped to the *G. undata* pseudo reference genome and the figure is not to scale, genic regions are exaggerated in size to easily locate on the chromosome map. Genes that contain both outstanding divergence and positive selection are hihglighted. b) Venn diagram displaying the number of concordant genes with outstanding divergence and significant positive selection, or, under divergent selection.

Figure 5 shows the overall window frequency of Tajima's D statistics for the five populations, where the collective majority had negative values. Figure 6 shows genes that contained d_N_/d_S_ values under significant positive selection (>3 SD) and the overall gene frequency of d_N_/d_S_ statistics for the five populations. The frequency of genes with outlier d_N_/d_S_ values per population is shown in Fig. 7; 68 genes containing outlier d_N_/d_S_ sites were considered to be under significant positive selection. Genomic locations of all genes with outstanding divergence (F_ST_ > 3 SD) and significant positive selection (d_N_/d_S_ > 3 SD) were mapped to the chromosomal scaffolds of the *G. undata* pseudo reference genome and are displayed in Fig. 8a. Finally, the number of concordant genes with outstanding divergence and significant positive selection are displayed in Fig. 8b and the known descriptions of these genes are displayed in Table 2.

**Table 2:** Descriptions of the two genes with statistics displaying divergent selection and which chromosome the gene is found on

Divergent selection gene	Chromosome	Description
LOC105048141	NC_025999.1	Gibberellin 2-beta-dioxygenase 1-like
LOC105051559	NC_026001.1	Probable DEAD-box ATP-dependent RNA helicase

## Discussion

In this work, we present an in-depth look at the genomic regions that are likely underlying the divergence of palm populations in the *G. undata* complex from targeted sequencing of 156 individuals sampled in the Northern Andes of Colombia. We propose that the *G. undata* complex is undergoing allopatric selection even in populations that are geographically sympatric, albeit located along an elevational range and isolated by relief. These genetic populations have locally undergone population expansions which further enhances genetic drift and matches with the high abundance but localized population patches observed in the field. Besides allopatry and drift by population expansion, we found a set of nuclear regions that are under divergent selection and can be implicated in the hyper-cryptic radiation as described by Olivares et al. (2024). Some of these genic regions potentially play functional roles in seasonal phenology and environmental response.

### There Are Likely Four to Five Populations of the *G. undata* Complex in the Northern Colombian Andes and Two to Three of Them Are Sympatric

Our results revealed one additional population in the Northern Colombian Andes compared to the previous study by Sanín et al. (2022) and one less population in this region when compared to Olivares et al. (2024). Discrepancies in the number of populations can be a result of the differences in sample sizes per site and recovered variants; here, we recovered 212,888 variable sites across 4,184 genic and nongenic regions, compared to 12,750 in Sanín et al. (2022); and used 156 individuals compared to 419 in Olivares et al. (2024), which studied at the continental level. Nevertheless, our population structure supports Sanín et al. (2022), with divergence of populations in concordance with Pliocene strike-slip faulting, splitting the Magdalena, Orinoco, and Amazonian drainages from the Western and Central Cordilleras (Sanín et al. 2022). The principal component analyses (PCAs) display much heterozygosity and genetic variation in the CM population (Fig. S1), which corresponds to the Colombian Massif.

### Allopatric Selection Occurs Even Among Sympatric Populations

Divergence with gene flow occurs when two populations undergo division that leads to reproductive isolation, and the possibility for speciation, while still allowing for gene flow to occur between the two populations (Harrison 2012). Historically, divergence with gene flow occurs under different levels of sympatry (Pinho and Hey 2010). We explored in detail the evolutionary scenarios between the WCC1 and WCC2 populations due to their sympatric geographic distribution in the Western and Central Cordilleras (Fig. S1). We expected to see high indications of divergence with gene flow occurring between these two populations, made evident by the number of windows with high F_ST_ and D_XY_ values and low Pi values.

Interestingly, we found more evidence for allopatric selection occurring between these two populations, due to the large number of windows with high F_ST_ values, moderate D_XY_ values, and low Pi values (Table 1). This is likely caused by the two populations occurring along an elevational gradient in the Western and Central Cordilleras of the Northern Colombian Andes (Fig. S3). Although there is some elevational overlap in the growing distribution of these two populations, significance statistics did reveal an elevational difference in means (Fig. 2). This fundamental role of elevational parapatry is in agreement with Olivares et al. (2024), where populations located in different regions of the Neotropics were geographically distant, despite grouping in the same clade, growing at the same elevation, and displaying morphological similarities (Olivares et al. 2024). In the sympatric Western and Central cordillera populations described here, geographically proximal individuals on different slopes and elevations grouped into different populations. In both instances described, there is evidence that supports the conclusion of gene flow being more constrained by topographic barriers, than geographic distance. The ability for elevational gradients to produce topographic barriers that lead to population divergence has also been studied in other regions of the Andean Mountain range, in populations of Neotropical birds (Benham and Witt 2016; Bertrand et al. 2016). Our results, together with those of Olivares et al. (2024), contribute to our understanding that elevational gradients are likely to play a role in the divergence of populations in the *G. undata* complex, especially those located in the Northern Colombian Andes. Furthermore, although two genetic groups are sympatric, the prevalence of the “allopatric scenario” through the genome suggest a recent secondary contact, probably linked to population expansion (Fig. 4), after a marked phase of allopatry favored by geographic and topographic isolation.

The WCC2 and EC populations were selected for pairwise comparison due to their geographic allopatry, where the WCC2 population is located on the Western and Central Cordilleras and the EC population is located on the Eastern Cordillera (Fig. S1). Given these parameters, we expected to see high amounts of allopatric selection and little evidence of gene flow (Shang et al. 2023). These expectations were met, as there were few windows displaying divergence with gene flow, and many under allopatric selection (Table 1). The EC and CM populations were also compared due to their geographic allopatry (Fig. S1). We expected to see high support for allopatric selection, and this expectation was met with the number of windows with high F_ST_ values, moderate D_XY_ values, and low Pi values (Table 1). Interestingly, there was also support for divergence with gene flow in this pairwise comparison, which is likely due to dispersal between the Eastern Cordillera and the Western and Central Cordilleras from the Colombian Massif. This corroborates the findings that the Colombian Massif (CM) population is likely serving as an intermediate population, acting as a proxy for gene flow to go around the Magdalena River valley.

### Recent Population Expansions and Selection in the *G. undata* Complex

The majority of Tajima's D values are negative at the window level for the five populations of the *G. undata* complex in the Northern Colombian Andes (Fig. 5, range of mean Tajima's D: −0.8953 to −0.1957), both seen as evolving neutrally and not (Fig. 5). These overall negative Tajima's D estimates were not affected by differences in population size and the defined population structure (Figs. S4 and S5). This abundance of rare alleles, likely representative of recent population expansions after population bottlenecks (Tajima 1989), is consistent with the “isolation-with-migration and growth” demographic models found for populations in the Western and Central cordilleras by Sanín et al. (2022). In the context of the topographically isolated Andean valleys, genetic differentiation may be driven by local adaptation and drift in expanding populations that undergo at least some phases of allopatry.

Throughout the five populations of the *G. undata* complex in the Northern Colombian Andes, we found many genes with d_N_/d_S_ values < 1, indicating large amounts of purifying selection, with few specific loci under positive selection (Fig. 6) (Nei and Gojobori 1986). This result may be due to the nature of the targeted capture genetic data, which consists mainly of genes with intentionally minimal neutral regions. In addition, between 30 and 45 genes per population contained d_N_/d_S_ values > 3 SD of the mean, indicating significant positive selection. Many of these genes are concordant between the populations (Fig. 7), suggesting that positive selection acted on these genes before the expansion of the *G. undata* complex in the Northern Colombian Andes. The discordance between genes under significant positive selection (d_N_/d_S_ > 3 SD) and with outstanding divergence (F_ST_ > 3 SD) between populations (Fig. 8) also suggests that most detected positive selection may have preceded expansion of the *G. undata* complex into the Northern Colombian Andes, hyper-cryptic radiation, and specific local adaptation in the different populations.

### Regions Under Divergent Selection Are Related to Temporal Phenology

Broadly, our findings show that selection and divergence are taking place in different regions of the *G. undata* complex genome, with little overlap of genes displaying both high divergence statistics (Hudson's F_ST_) and selection statistics (d_N_/d_S_) (Fig. 8a). LOC105051559 and LOC105048141, the two genes under divergent selection by outlier F_ST_ and d_N_/d_S_ statistics (Fig. 8b, Table 2), both play functional roles in phenology. LOC105051559 is a probable DEAD-box helicase. DEAD-box helicases regulate RNA processing and metabolism and can control gene expression that affects or influences plant growth and development (Tuteja et al. 2014), sometimes driven by abiotic stress (Macovei et al. 2012). Dependent upon environmental factors and abiotic stressors, these DEAD-box helicases could differ RNA regulation (Hao et al. 2022) among populations, through changes of seasonal patterns in expression. LOC105048141 is an ortholog for GA2ox2 in the highly studied model organism *Arabidopsis thaliana* (Teshome and Kebede 2021), and has the functional description of gibberellin 2-beta-dioxygenase 1-like. Genes in the Gibberellin-dioxygenase family contain the gibberellin hormone, and play functional roles in numerous regulatory processes, including stem elongation, germination, flower timing, and leaf and fruit senescence (Hedden and Sponsel 2015). Additionally, GA2ox2 mRNA has shown to change in response to variations in light environments, as well as differences in temperature (Provart et al. 2003). Collectively, these functional roles of LOC105048141 could also influence phenological variation in different populations of the *G. undata* complex exposed to different elevations and environments in the Northern Colombian Andes.

These genic descriptions and functions can shed light on potentially interesting regions of divergent selection in the *G. undata* complex. However, we cannot say with certainty whether these exact genes are undergoing divergent selection, or if they are a part of a larger selective sweep that was not sequenced in this study (made evident by genetic hitchhiking). Although these results should be interpreted with caution, these genes represent the first interesting candidate genes for phenotypic variation between divergent populations in the *G. undata* complex.

### Hyper-Cryptic Speciation in the Complex Is Driven by a Multitude of Factors, Not Solely Selection

In light of the measures of divergence and selection statistics for populations, the *G. undata* complex is undergoing speciation, although the extent of reproductive isolation between the groups is currently unknown. Our results support the findings of Olivares et al. (2024), which established that the *G. undata* complex is an example of a hyper-cryptic radiation, where divergence is occurring, and morphology has yet to be fixed (Olivares et al. 2024). This is supported by the Admixture results, where three of the five populations comprise individuals of both taxonomically identified species, *G. undata* and *G. orbignyana* (Fig. S1). Cryptic complexes are groups of species that contain individuals that are morphologically similar, despite being unable to interbreed in some instances. An example of this can be found in the frog genus *Eleutherodactylus*, where there are many cryptic species with varying morphological forms (Díaz et al. 2012). Although cryptic species have been studied in various organisms such as rotifers (Suatoni et al. 2006), fish (Adams et al. 2014), frogs (Díaz et al. 2012), and Neotropical plants (Vieu et al. 2023), it is still unclear how to categorize these species in biodiversity science (Fišer et al. 2018). Sympatric divergence has occurred due to other factors in the genus, such as reproductive isolation (Borchsenius et al. 2016), that were not explored here due to lack of phenotypic data. However, phenological patterns are not important for reproductive isolation in other variable forms of *Geonoma*, such as *Geonoma cuneata* H. Wendl. ex Spruce (Ley-López et al. 2024), so patterns can be idiosyncratic and not generalizable between species or populations. Hence, for now, we consider that the *G. undata* complex is an example of cryptic species complex undergoing speciation, with the populations at different locations on the speciation continuum.

### Main Conclusions and Future Directions

Our study shows that cryptic divergence in the *G. undata* complex is driven by several factors, primarily involving (i) geographical and elevational isolation (although not always persistent), (ii) genetic drift by population expansion, and (iii) selection. Our findings corroborate the results of Sanín et al. (2022) and Olivares et al. (2024), and expand by establishing the roles of drift and divergent selection compounded with allopatry and parapatry across elevational gradients to generate genetic and morphological diversity.

To further understand the genetic mechanisms that are driving divergence in the *G. undata* complex, future studies should generate chromosome-level reference genomes, whole transcriptomes, and whole epigenomes to provide refined insight and high resolution to genomic drivers of divergence and speciation in present-day regimes. Some of the genes under selection are shared between populations meaning they possibly predate population divergence. These new molecular tools would also allow for research on differentially expressed genes among populations, especially with those playing a role in seasonal phenology and response to environmental stress. This is particularly true for individuals in populations that are genetically close, morphologically distinct and geographically sympatric in the Western and Central Cordilleras. Additionally, future population-level studies should be conducted using whole genome resequencing methods, which will yield large amounts of neutral regions in the genome that can be used to infer a more refined demographic history of the *G. undata* complex.

## Materials and Methods

### Sampling, DNA Sequencing, Calling Single Nucleotide Polymorphisms, and Variant Call Format Filtering

The genetic data utilized here is a portion of the data utilized in Olivares et al. (2024), where population structure of the *G. undata* complex across the entire range of its growing distribution was explored, from Mexico to Bolivia. Here, we focus on understanding drivers of genomic divergence for individuals that comprise the Northern Colombian Andes due to their population sympatry and the intricate topography of this region. Leaf sampling took place in 26 localities of the Northern Andes, including three mountain ranges in Colombia (Eastern, Central, and Western Cordilleras), and the Sierra Nevada de Santa Marta mountain of the Caribbean region. A total of 156 individuals of the *Geonoma undata* complex were sequenced, comprising of 52 individuals of *G. orbignyana* morphology and 104 individuals of *G. undata* morphology, plus two individuals of *Geonoma interrupta* (Ruiz & Pav.) Mart. from Sanín et al. (2022) as outgroups for d_N_/d_S_ statistics. During field work, leaf samples were collected and stored in silica gel to preserve DNA and were then deposited in the collections at Universidad CES while DNA was extracted at Universidad CES. DNA was extracted using the DNeasy Plant Mini Kit (Qiagen) and manufacturing protocol. DNA fragmentation, dual-indexed library preparation, and targeted sequence capture were all conducted at the University of Fribourg (Fribourg, Switzerland) following de La Harpe et al. (2019).

The samples were sequenced using a targeted sequencing approach with the Arecaceae-specific PopcornPalm kit (de La Harpe et al. 2019). This kit comprises of 4,184 genomic regions, where 4,051 regions are genic-coding, and 133 regions are nongenic putatively neutral regions (de La Harpe et al. 2019). Raw genomic data are publicly available on NCBI (NCBI bioprojects PRJNA482221, PRJNA541164, and PRJNA707300, from de La Harpe et al. (2019), Loiseau et al. (2019), and Sanín et al. (2022).

We called single nucleotide polymorphisms (SNPs) for the 4,184 target regions and their surrounding 400 bp regions using Haplotype caller in GATK v3.6 (McKenna et al. 2010) and the EMIT_ALL_SITES option to obtain variant and nonvariant sites. Then, the output variant call format (VCF) file was filtered using VCFtools v0.1.13 (Danecek et al. 2011) and the following parameters: minimum quality score of 20, minimum sequencing depth of 15 and maximum sequencing depth of 50 per sample, and maximum of 10% of missing data allowed and retaining only two alleles per site.

### Genetic Structure and Population Characterizing (PCAs and Admixture)

Initial exploration of the data began by calculating 20 principal components (PCs) with Plink v1.9 beta (Purcell et al. 2007) where linkage pruning was conducted with a window size of 50 kb, step size of ten variants, and 0.1 as the *r*^2^ threshold, and indels were removed beforehand using VCFtools v0.1.13 (Danecek et al. 2011). The percent variance explained for each PC was calculated in R v4.2.2 (R Core Team 2022), and PCs 1 to 3 were visualized in R using the *tidyverse* package (Wickham et al. 2019). PCs were colored and shaped by different combinations of taxonomic identification and later, population.

Genetic groups were identified using Admixture v1.3 (Alexander et al. 2009) cross-validation once linkage pruning was conducted and the VCF was filtered of loci where >99.9% of genotypes were missing. Ancestry coefficients for K = 1 to 15 were calculated and visualized to assess K values. PCAs, Admixture, and cross-validation results were visualized in R (R Core Team 2022) using the *tidyverse* (Wickham et al. 2019) and *ggthemes* (Arnold 2024) packages. Geographic distribution of populations was also visualized in R (R Core Team 2022) using the *elevatr* (Hollister et al. 2023), *rgeoboundaries* (Runfola et al. 2020), *tidyverse* (Wickham et al. 2019), *ggspatial* (Dunnington 2023), and *ggsn* (Baquero 2017) packages.

### Elevational Exploration of Populations

To explore if there were any statistically significant differences among populations by elevation, R packages *elevatr* (Hollister et al. 2023), *sf* (Pebesma 2018; Pebesma and Bivand 2023), *raster* (Hijmas 2024), *rgdal* (Bivand et al. 2023), and *sp* (Pebesma and Bivand 2005; Bivand et al. 2013) were used to plot *G. undata* complex individuals over an elevational raster. Elevations for each sample were extracted and visualized with the R packages *tidyverse* (Wickham et al. 2019), *FSA* (Bogetoft and Otto 2024), *rstatix* (Kassambara 2023b), and *ggpubr* (Kassambara 2023a). Population pairs were compared using a Kruskal–Wallis test followed by a Dunn test with a Bonferroni correction for *P*-values of multiple pairwise comparisons.

### Population Diversity and Divergence Statistics (Pi, D_XY_, and F_ST_)

We estimated absolute and relative divergence (D_XY_ and Hudson's F_ST_), for all ten pairwise comparisons of the five populations using a 10,000 bp sliding window approach and the software pixy v1.2.7.betal (Korunes and Samuk 2021). To measure within-group nucleotide diversity (Pi), five separate VCF files were parsed, one per population, and we utilized a 10,000 bp sliding window approach with the software pixy v1.2.7.betal (Korunes and Samuk 2021). Population diversity and divergence statistics were summarized and visualized in R (R Core Team 2022) using the *tidyverse* package (Wickham et al. 2019).

Genetic windows with outstanding divergence, or outlier F_ST_ statistics, were calculated and identified using standard deviation from the mean (SD). Windows with F_ST_ values greater than 3 standard deviations from the mean were considered outliers. Windows with outlier F_ST_ values were then mapped to their corresponding genic regions and functions were identified using an in-house Python script.

To understand the behavior of D_XY_, Hudson's F_ST_, and Pi between the different populations, dot plots and regression analyses were conducted for the three scenarios: (i) populations that are sympatric in the Western and Central cordilleras, (ii) populations that are allopatric with the Western and Central cordilleras and Eastern cordillera, and (iii) populations that are allopatric with the Western and Central cordilleras and Colombian Massif. This understanding of summary statistic behavior can help identify different evolutionary scenarios to link genomic variation to environmental conditions, as described in Shang et al. (2023) (Han et al. 2017; Irwin et al. 2018; Shang et al. 2023). D_XY_ and F_ST_ were used as pairwise relative statistics, and Pi was averaged between the two populations in the pairwise comparisons. Values were calculated for 1, 2, and 3 standard deviations from the mean for F_ST_, D_XY_, and Pi, and these values were used to identify windows displaying different evolutionary scenarios following Shang et al. (2023). Models were defined as follows: F_ST_ > 2 SD, D_XY_ > 3 SD, and Pi < 3 SD = *divergence with gene flow*; F_ST_ > 2 SD, D_XY_ < 3 SD, and Pi < 1 SD = *allopatric selection*; F_ST_ > 1 SD, and D_XY_ and Pi < 1 SD = *recurrent selection*; F_ST_ < 3 SD, and D_XY_ and Pi > 3 SD = *balancing selection* (Shang et al. 2023). D_XY_ and Pi were adjusted to a linear model, and the correlation coefficients (rho), based on Spearman methods for all three pairwise comparisons. These analyses were run using an in-house R script using the *tidyverse* (Wickham et al. 2019), *ggpubr* (Kassambara 2023a), and *ggnewscale* (Campitelli 2024) packages.

### Population Dynamics and Selection Statistics (Tajima's D and d_N_/d_S_)

To compare observed nucleotide diversity with expected and infer genome wide trends of recent population history, Tajima's D statistics were calculated for all five populations using VCFtools v0.1.13 (Danecek et al. 2011) and a sliding window approach (window size of 10,000 bp). Then, all Tajima's D statistics were visualized and summarized in R (R Core Team 2022) using the *tidyverse* package (Wickham et al. 2019). Tajima's D is a selection statistic that identifies regions in the genome that are neutral (Tajima 1989), where genomic windows with Tajima's D values that are >2 and <−2 are typically viewed as not evolving neutrally (Tajima 1989; Eckshtain-Levi et al. 2018). Genomic windows with values > 2 are thought to be undergoing balancing selection, and genomic windows with <−2 are usually seen as undergoing positive selection (Tajima 1989; Eckshtain-Levi et al. 2018). Along with selection occurring throughout the genome, Tajima's D statistics can be used to infer how population demographics have changed throughout time (Tajima 1989).

To incorporate an avenue for identifying specific loci under significant positive selection, d_N_/d_S_ statistics for each population were calculated using two individuals of *G. interrupta* as outgroup. We approximated all amino acids to be 4-fold degenerate, separating non-synonymous sites corresponding to the 1st and 2nd base pair and synonymous sites corresponding to the 3rd base pair in the codon. Only sites covered with a maximum of 50% missing data were retained for the analyses, and the number of substitutions between each population and the outgroup species was calculated using the R package *PopGenome* (Pfeifer et al. 2014). Only genes with at least one synonymous and one non-synonymous fixed site to the outgroup were kept for the d_N_/d_S_ calculation. d_N_ was calculated as the ratio of the number of non-synonymous fixed sites to the outgroup over the total number of covered non-synonymous sites; d_S_ was calculated as the ratio of the number of synonymous fixed sites to the outgroup over the total number of covered synonymous sites. All d_N_/d_S_ statistics were visualized and summarized in R (R Core Team 2022) using the *tidyverse* package (Wickham et al. 2019). The d_N_/d_S_ statistic is a measure of synonymous to non-synonymous substitutions in a given protein coding gene (Kimura 1977) and can be used to understand selection occurring in populations (Kryazhimskiy and Plotkin 2008). Broadly, genes with d_N_/d_S_ statistics < 1 are undergoing purifying selection, genes with d_N_/d_S_ values at approximately 1 are neutrally evolving, and d_N_/d_S_ values > 1 are undergoing positive selection (Kryazhimskiy and Plotkin 2008). Genes with d_N_/d_S_ statistics > 3 SD from the mean were considered to be under outstanding positive selection.

To identify concordant genic regions with outstanding divergence and significant positive selection, the genes containing outliers by 3 standard deviations from the mean for Hudson's F_ST_ and d_N_/d_S_ statistics were summarized and visualized in R (R Core Team 2022) using the *tidyverse* (Wickham et al. 2019), *ggVennDiagram* (Gao et al. 2021), *ggnewscale* (Campitelli 2024), *ggthemes* (Arnold 2024), and *ggvenn* (Yan 2023) packages.

## Supplementary Material

evaf130_Supplementary_Data

## Data Availability

The 156 samples used for this manuscript are publicly available on NCBI (NCBI bioprojects PRJNA482221, PRJNA541164, PRJNA707300).

## References

[evaf130-B1] Adams M, Raadik TA, Burridge CP, Georges A. Global biodiversity assessment and hyper-cryptic species complexes: more than one species of elephant in the room? Syst Biol. 2014:63:518–533. 10.1093/sysbio/syu017. 24627185

[evaf130-B2] Alexander DH, Novembre J, Lange K. Fast model-based estimation of ancestry in unrelated individuals. Genome Res. 2009:19:1655–1664. 10.1101/gr.094052.109. 19648217 PMC2752134

[evaf130-B3] Arnold JB . *ggthemes: extra themes, scales and geoms for “ggplot2”,* R Package Version (5.1.0). 2024. 10.32614/CRAN.package.ggthemes.

[evaf130-B4] Baquero OS . *North symbols and scale bars for maps created with “ggplot2” or “ggmap”.* R package version 0.4.0. 2017. https://CRAN.R-project.org/package=ggsn.

[evaf130-B5] Benham PM, Witt CC. The dual role of Andean topography in primary divergence: functional and neutral variation among populations of the hummingbird, *Metallura tyrianthina*. BMC Evol Biol. 2016:16:22. 10.1186/s12862-016-0595-2. 26801894 PMC4724075

[evaf130-B6] Bertrand JAM, et al The role of selection and historical factors in driving population differentiation along an elevational gradient in an island bird. J Evol Biol. 2016:29:824–836. 10.1111/jeb.12829. 26779843

[evaf130-B7] Bivand R, Keitt T, Rowlingson B. rgdal: bindings for the “Geospatial” data abstraction library. 2023. https://cran.r-project.org/src/contrib/Archive/rgdal/.

[evaf130-B8] Bivand RS, Pebesma E, Gomex-Rubio V. Applied spatial data analysis with {R}. 2nd ed. Springer; 2013. http://rgdal.r-forge.r-project.org, https://gdal.org, https://proj.org, https://rforge.r-project.org/projects/rgdal/.

[evaf130-B9] Bogetoft P, Otto L. *Benchmarking with DEA and SFA* (version 0.32). 2024.

[evaf130-B10] Borchsenius F, Lozada T, Knudsen JT. Reproductive isolation of sympatric forms of the understorey palm *Geonoma Macrostachys* in Western Amazonia. Bot J Linn Soc. 2016:182:398–410. 10.1111/boj.12428.

[evaf130-B11] Campitelli E . ggnewscale: multiple fill and colour scales in “ggplot2” (version 0.4.10.9). 2024. https://cran.r-project.org/web/packages/ggnewscale/readme/README.html.

[evaf130-B12] Danecek P, et al The variant call format and VCFtools. Bioinformatics. 2011:27:2156–2158. 10.1093/bioinformatics/btr330. 21653522 PMC3137218

[evaf130-B13] de La Harpe M, et al A dedicated target capture approach reveals variable genetic markers across micro- and macro-evolutionary time scales in palms. Mol Ecol Resour. 2019:19:221–234. 10.1111/1755-0998.12945. 30240120

[evaf130-B14] Díaz LM, Hedges SB, Schmid M. A new cryptic species of the genus *Eleutherodactylus* (Amphibia: Anura: Eleutherodactylidae) from Cuba. Zootaxa. 2012:3220:44–60. 10.11646/zootaxa.3220.1.3.

[evaf130-B15] Dunnington D . ggspatial: spatial data framework for ggplot2. 2023. https://cran.r-project.org/web/packages/ggspatial/index.html.

[evaf130-B16] Eckshtain-Levi N, Weisberg AJ, Vinatzer BA. The population genetic test Tajima’s D identifies genes encoding pathogen-associated molecular patterns and other virulence-related genes in *Ralstonia solanacearum*. Mol Plant Pathol. 2018:19:2187–2192. 10.1111/mpp.12688. 29660239 PMC6638162

[evaf130-B17] Fišer C, Robinson CT, Malard F. Cryptic species as a window into the paradigm shift of the species concept. Mol Ecol. 2018:27:613–635. 10.1111/mec.14486. 29334414

[evaf130-B18] Galeano G, Bernal R. *Palmas de Colombia. Guía de campo.* (Editorial Universidad Nacional de Colombia, Ed.). Instituto de Ciencias Naturales-Universidad Nacional de Colombia; 2010.

[evaf130-B19] Galtier N . Delineating species in the speciation continuum: a proposal. Evol Appl. 2019:12:657–663. 10.1111/eva.12748. 30976300 PMC6439491

[evaf130-B20] Gao CH, Yu G, Cai P. ggVennDiagram: an intuitive, easy-to-use, and highly customizable R package to generate Venn diagram. Front Genet. 2021:12:706907. 10.3389/fgene.2021.706907. 34557218 PMC8452859

[evaf130-B21] Han F, et al Gene flow, ancient polymorphism, and ecological adaptation shape the genomic landscape of divergence among Darwin’s finches. Genome Res. 2017:27:1004–1015. 10.1101/gr.212522.116. 28442558 PMC5453315

[evaf130-B22] Hao LL, et al Comprehensive comparative analysis and expression profiles and effects on physiological response of DEAD-box RNA helicase genes in *Lumnitzera littorea* (Jack) Voigt under cold stress. J Plant Interact. 2022:17:595–607. 10.1080/17429145.2022.2074158.

[evaf130-B23] Harrison RG . The language of speciation. Evolution. 2012:66:3643–3657. 10.1111/j.1558-5646.2012.01785.x. 23206125

[evaf130-B24] Hedden P, Sponsel V. A century of gibberellin research. J Plant Growth Regul. 2015:34:740–760. 10.1007/s00344-015-9546-1. 26523085 PMC4622167

[evaf130-B25] Henderson A . A revision of Geonoma (Arecaceae). Phytotaxa. 2011:17:1–271. 10.11646/phytotaxa.17.1.1.

[evaf130-B26] Hijmas RJ . raster: geographic data analysis and modeling. 2024. https://cran.r-project.org/web/packages/raster/index.html.

[evaf130-B27] Hollister J, Beck MW, Johnson M. elevatr: access elevation data from various APIs (version 0.99.0). 2023. https://cran.r-project.org/web/packages/elevatr/index.html.

[evaf130-B28] Irwin DE, et al A comparison of genomic islands of differentiation across three young avian species pairs. Mol Ecol. 2018:27:4839–4855. 10.1111/mec.14858. 30187980

[evaf130-B29] Kassambara A . ggpubr: “ggplot2” based publication ready plots (R package version 0.6.0). 2023a. https://cran.r-project.org/web/packages/ggpubr/index.html.

[evaf130-B30] Kassambara A . rstatix: pipe-friendly framework for basic statistical tests (version 0.7.2). 2023b. https://cran.r-project.org/web/packages/rstatix/index.html.

[evaf130-B31] Kimura M . Preponderance of synonymous changes as evidence for the neutral theory of molecular evolution. Nature. 1977:267:275–276. 10.1038/267275a0. 865622

[evaf130-B32] Knudsen JT . Variation in floral scent composition within and between populations of *Geonoma macrostachys* (Arecaceae) in the western Amazon. Am J Bot. 2002:89:1772–1778. 10.3732/ajb.89.11.1772. 21665604

[evaf130-B33] Korunes KL, Samuk K. pixy: unbiased estimation of nucleotide diversity and divergence in the presence of missing data. Mol Ecol Resour. 2021:21:1359–1368. 10.1111/1755-0998.13326. 33453139 PMC8044049

[evaf130-B34] Kryazhimskiy S, Plotkin JB. The population genetics of dN/dS. PLoS Genet. 2008:4:e1000304. 10.1371/journal.pgen.1000304. 19081788 PMC2596312

[evaf130-B35] Ley-López JM, Alvarado CM, Valerio ER, Wawrzyniak MK, Chmielarz P. Phenological patterns from two sympatric subspecies of the palm *Geonoma cuneata* (H. Wendl. Ex Spruce) and their gall inductor Contarinia geonomae (Gagné). Dendrobiology. 2024:91:70–84. 10.12657/denbio.091.006.

[evaf130-B36] Loiseau O, et al Targeted capture of hundreds of nuclear genes unravels phylogenetic relationships of the diverse neotropical palm tribe Geonomateae. Front Plant Sci. 2019:10:864. 10.3389/fpls.2019.00864. 31396244 PMC6640726

[evaf130-B37] Macovei A, Vaid N, Tula S, Tuteja N. A new DEAD-box helicase ATP-binding protein (OsABP) from rice is responsive to abiotic stress. Plant Signal Behav. 2012:7:1138–1143. 10.4161/psb.21343. 22899052 PMC3489646

[evaf130-B38] McKenna A, et al The genome analysis toolkit: a MapReduce framework for analyzing next-generation DNA sequencing data. Genome Res. 2010:20:1297–1303. 10.1101/gr.107524.110. 20644199 PMC2928508

[evaf130-B39] Nei M, Gojobori T. Simple methods for estimating the numbers of synonymous and nonsynonymous nucleotide substitutions. Mol Biol Evol. 1986:3:418–426. 10.1093/oxfordjournals.molbev.a040410. 3444411

[evaf130-B41] Olivares I, et al Hyper-cryptic radiation of a tropical montane plant lineage. Mol Phylogenet Evol. 2024:190:107954. 10.1016/j.ympev.2023.107954. 37898295

[evaf130-B43] Pebesma E . Simple features for R: standardized support for spatial vector data. R J. 2018:10:438–446. 10.32614/RJ-2018-009.

[evaf130-B44] Pebesma E, Bivand R. Spatial data science: with applications in R. Chapman and Hall/CRC; 2023.

[evaf130-B45] Pebesma EJ, Bivand R. Classes and methods for spatial data in {R}. R News. 2005:5:9–13. ISSN 1609-3631.

[evaf130-B46] Pfeifer B, Wittelsbürger U, Ramos-Onsins SE, Lercher MJ. PopGenome: an efficient Swiss army knife for population genomic analyses in R. Mol Biol Evol. 2014:31:1929–1936. 10.1093/molbev/msu136. 24739305 PMC4069620

[evaf130-B47] Pinho C, Hey J. Divergence with gene flow: models and data. Annu Rev Ecol Evol Syst. 2010:41:215–230. 10.1146/annurev-ecolsys-102209-144644.

[evaf130-B48] Provart NJ, et al Gene expression phenotypes of Arabidopsis associated with sensitivity to low temperatures. Plant Physiol. 2003:132:893–906. 10.1104/pp.103.021261. 12805619 PMC167029

[evaf130-B49] Purcell S, et al PLINK: a tool set for whole-genome association and population-based linkage analyses. Am J Hum Genet. 2007:81:559–575. 10.1086/519795. 17701901 PMC1950838

[evaf130-B50] R Core Team . R: a language and environment for statistical computing. R Foundation for Statistical Computing; 2022.

[evaf130-B51] Rodríguez-Buriticá S, Orjuela MA, Galeano G. Demography and life history of *Geonoma orbignyana*: an understory palm used as foliage in Colombia. For Ecol Manage. 2005:211:329–340. 10.1016/j.foreco.2005.02.052.

[evaf130-B52] Runfola D, et al GeoBoundaries: a global database of political administrative boundaries. PLoS One. 2020:15:e0231866. 10.1371/journal.pone.0231866. 32330167 PMC7182183

[evaf130-B53] Sanín MJ, et al Geogenomics of montane palms points to Miocene–Pliocene Andean segmentation related to strike-slip tectonics. J Biogeogr. 2022:49:1711–1725. 10.1111/jbi.14327.

[evaf130-B54] Shang H, et al Drivers of genomic landscapes of differentiation across a Populus divergence gradient. Mol Ecol. 2023:32:4348–4361. 10.1111/mec.17034. 37271855

[evaf130-B55] Stankowski S, Ravinet M. Defining the speciation continuum. Evolution. 2021:75:1256–1273. 10.1111/evo.14215. 33754340

[evaf130-B56] Stropp J, Ladle RJ, Emilio T, Lessa T, Hortal J. Taxonomic uncertainty and the challenge of estimating global species richness. J Biogeogr. 2022:49:1654–1656. 10.1111/jbi.14463.

[evaf130-B57] Suatoni E, Vicario S, Rice S, Snell T, Caccone A. An analysis of species boundaries and biogeographic patterns in a cryptic species complex: the rotifer—*Brachionus plicatilis*. Mol Phylogenet Evol. 2006:41:86–98. 10.1016/j.ympev.2006.04.025. 16815046

[evaf130-B58] Tajima F . Statistical method for testing the neutral mutation hypothesis by DNA polymorphism. Genetics 1989:123:585–595. 2513255 10.1093/genetics/123.3.585 PMC1203831

[evaf130-B59] Teshome S, Kebede M. Analysis of regulatory elements in GA2ox, GA3ox and GA20ox gene families in *Arabidopsis thaliana*: an important trait. Biotechnol Biotechnol Equip. 2021:35:1603–1612. 10.1080/13102818.2021.1995494.

[evaf130-B60] Tuteja N, Tarique M, Banu MSA, Ahmad M, Tuteja R. *Pisum sativum* p68 DEAD-box protein is ATP-dependent RNA helicase and unique bipolar DNA helicase. Plant Mol Biol. 2014:85:639–651. 10.1007/s11103-014-0209-6. 24908423

[evaf130-B61] Vieu JC, Koubínová D, Grant JR. Population genetic structure and diversity of cryptic species of the plant genus *Macrocarpaea* (Gentianaceae) from the Tropical Andes. Plants. 2023:12:1710. 10.3390/plants12081710. 37111932 PMC10145315

[evaf130-B63] Wickham H, et al Welcome to the tTidyverse. J Open Source Softw. 2019:4:1686. 10.21105/joss.01686.

[evaf130-B64] Yan L . ggvenn: Draw a Venn diagram by “ggplot2” (version 0.1.10). 2023. https://cran.r-project.org/web/packages/ggvenn/readme/README.html.

