# Correction to: Divergence and Selection in a Cryptic Species Complex (*Geonoma undata*: Arecaceae) in the Northern Andes of Colombia

**DOI:** 10.1093/gbe/evag006

**Published:** 2026-01-19

This is a correction to: Carmen P Webster, Margot Paris, Ingrid Olivares, Martin F Wojciechowski, Michael Kessler, María José Sanín, Divergence and Selection in a Cryptic Species Complex (*Geonoma undata*: Arecaceae) in the Northern Andes of Colombia, *Genome Biology and Evolution*, Volume 17, Issue 7, July 2025, https://doi.org/10.1093/gbe/evaf130

The following changes have been made to the originally published manuscript.

The details of the fourth affiliation have been changed to read: “^4^Department of Systematic and Evolutionary Botany, University of Zurich, Zurich, Switzerland” instead of: “^4^Department for Biodiversity and Environment Research, University College London, London, UK”.

