# Supplementary material for: Divergence and Selection in a Cryptic Species Complex (Geonoma undata: Arecaceae) in the Northern Andes of Colombia
Source: Genome Biol Evol. 2025 Jul 22;17(7):evaf130. doi: 10.1093/gbe/evaf130 (PMC12279445; doi:10.1093/gbe/evaf130)
Supplement: evaf130_Supplementary_Data [file evaf130_supplementary_data.pdf]

Population characterization, structure, and geographic distribution

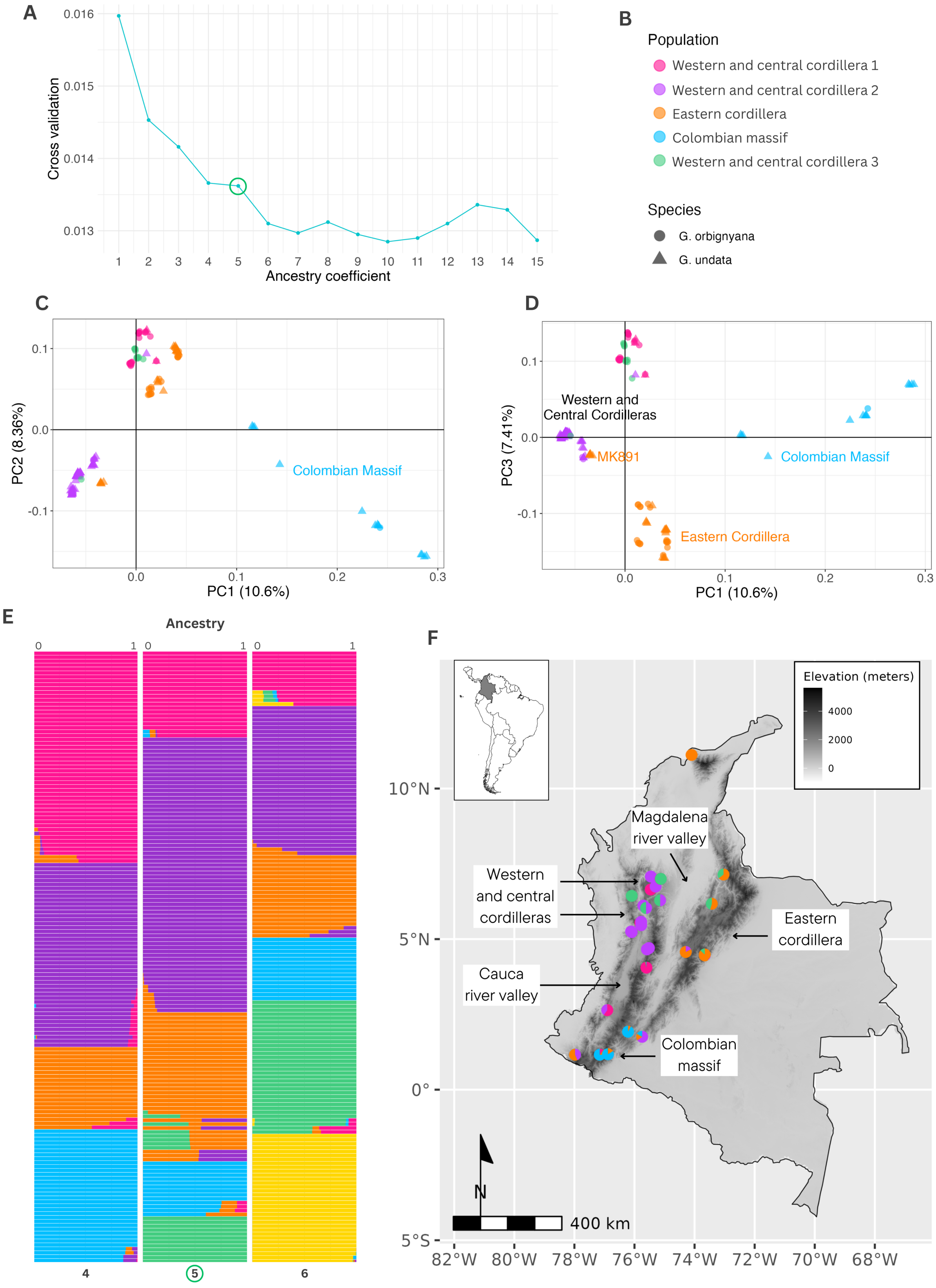

Suppl. Figure S1: Broad genetic characterization of the *G. undata* complex in the Northern Colombian Andes. (A) Cross validation statistics for ancestry coefficients of K=1-15. (B) Legend for PCAs, where individuals are colored by population for K=5, shaped by previous taxonomic identification, and labeled by geographic location. (C-D) Principal Component Analyses displaying the genetic structure of PC axes 1-3 for K=5. (E) Populations of pure and admixed individuals identified by Admixture K= 6, 5, and 4. K=5 is denoted by a green circle; the ancestry coefficient that downstream analyses were conducted with. (F) Map of Colombia on an elevation raster that displays the distribution of individuals colored by cumulative ancestry at each geographic locality for K=5. The darker the shade of grey, the higher the elevation.

Summary statistics plot for WCC1 vs. WCC2 no evolutionary scenarios

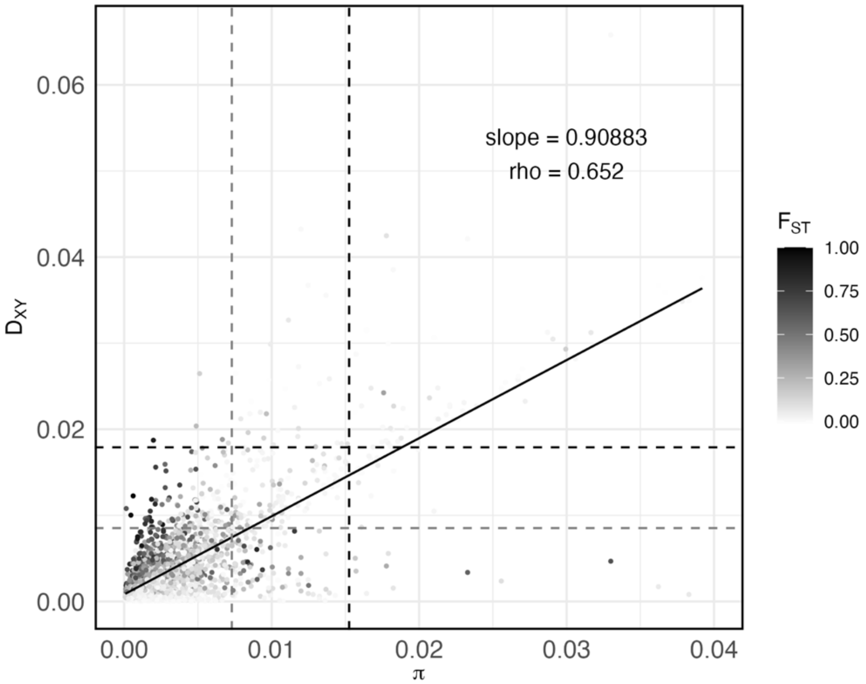

Suppl. Figure S2: Plotted summary statistics for the WCC1 vs. WCC2 pairwise comparison with a regression line in solid black. Absolute divergence, D_{XY}, is plotted on the y-axis, and averaged pairwise nucleotide diversity, Pi, is plotted on the x-axis. The upper boundary for both D_{XY} and Pi is plotted on the dashed black line and is representative of three standard deviations from the mean for both statistics. The lower boundary for both D_{XY} and Pi is plotted on the dashed gray line and is representative of one standard deviation from the mean for both statistics. The plotted points are shaded by their F_{ST} values, where a value of 1 is black and a value of 0 is white.

Parapatric speciation in the Western and Central Cordillera complex

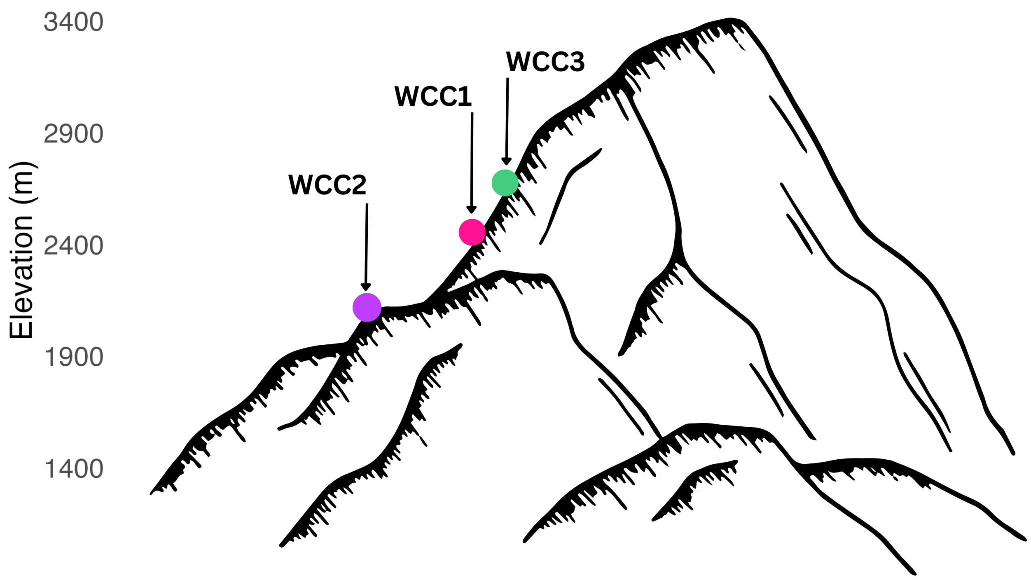

Suppl. Figure S3. Illustration displaying elevational relief leading to parapatric speciation in the *G. undata* complex. The mountains represent the Western and Central cordillera complex, where the WCC1, WCC2, and WCC3 populations are geographically proximal albeit elevationally segregated. The pink dot represents the population elevation mean for WCC1 population, the purple dot is mean elevation for the WCC2 population, and the green dot is mean elevation for the WCC3 population.

Tajima's D by consistent population sizes and K=2

Tajima's D statistic is sensitive to population size and structure (Moeller et al., 2007). To assess that the difference in sampling size between our 5 populations does not affect Tajima's D estimations, we additionally estimated Tajima's D using a subset of 10 individuals of pure ancestry from each of the 5 populations (Suppl. Figure 3). The average Tajima's D values remain negative for all 5 populations when using a subset of 50 samples out of the 156 examined in this article. Similar to the results presented in the main text, the orange (EC) and the purple (WCC2) populations show the lowest Tajima's D values on average, and the green (WCC3) population show the highest values, although still slightly negative on average. We compared the Tajima's D estimates at the window level between the main and the subset datasets and found that the Pearson correlation coefficient for each of the 5 populations range of from 0.398 to 0.571.

To further address the sensitivity of Tajima's D statistics to population structure, Tajima's D statistics were estimated considering the Admixture groups for K=2, which represent only a small fraction of the population structure present in our Colombian sample (Suppl. Figure 4). Admixture group 1 and group 2 contained 75 and 81 samples respectively. Tajima's D means and overall distribution for these two Admixture groups were negative and similar to the results

obtained with the 5 populations presented in the main text of this study. Taken together, these results suggest that the differences in population size between our populations and the defined population structure did not affect the overall negative Tajima's D estimates presented in the main text of this article.

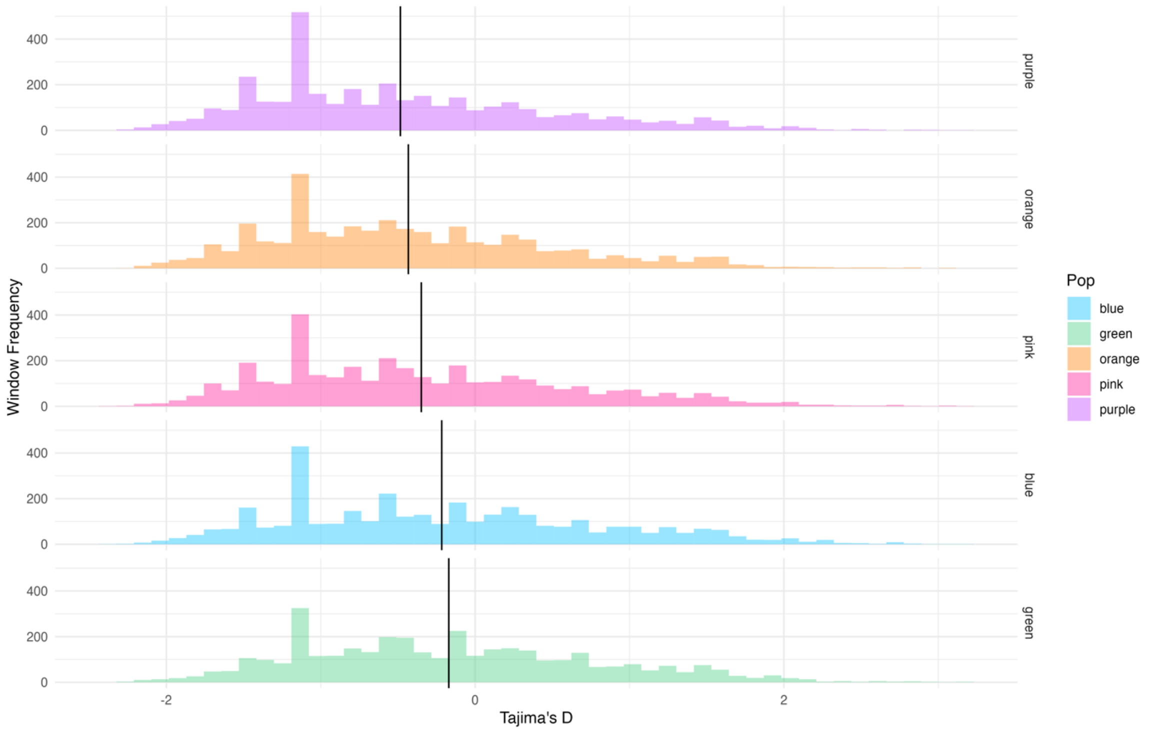

Suppl. Figure S4: Tajima's D for only 10 individuals from each of the 5 populations from Suppl. Figure 1; x-axis: Tajima's D values; y-axis: frequency; vertical black bars indicate the mean Tajima's D value.

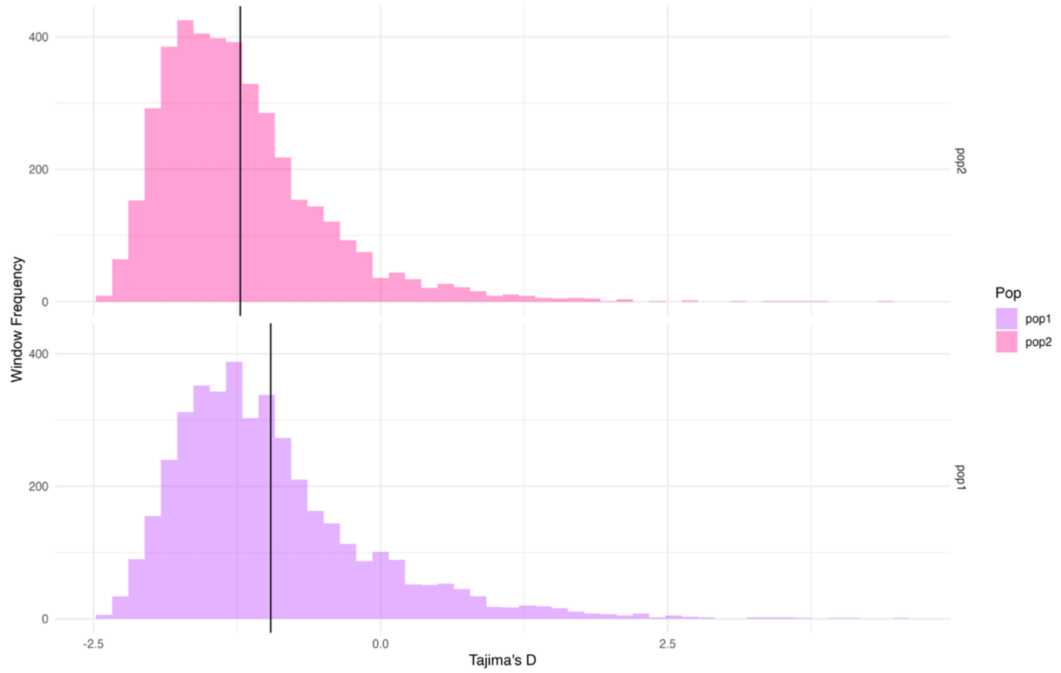

Suppl. Figure S5: Tajima’s D for all 156 individuals distributed by ancestry coefficient of K=2; x-axis: Tajima’s D values; y-axis: frequency; vertical black bars indicate the mean Tajima’s D value.
